# Tamper-Resistant Mobile Health Using Blockchain Technology

**DOI:** 10.2196/mhealth.7938

**Published:** 2017-07-26

**Authors:** Daisuke Ichikawa, Makiko Kashiyama, Taro Ueno

**Affiliations:** ^1^ Sustainable Medicine, Inc. Tokyo Japan; ^2^ Institute of Neuropsychiatry Seiwa Hospital Tokyo Japan; ^3^ Graduate School of Science Department of Biomolecular Science Toho University Chiba Japan

**Keywords:** telemedicine, electronic health records, sleep, cognitive therapy, computer security

## Abstract

**Background:**

Digital health technologies, including telemedicine, mobile health (mHealth), and remote monitoring, are playing a greater role in medical practice. Safe and accurate management of medical information leads to the advancement of digital health, which in turn results in a number of beneficial effects. Furthermore, mHealth can help lower costs by facilitating the delivery of care and connecting people to their health care providers. Mobile apps help empower patients and health care providers to proactively address medical conditions through near real-time monitoring and treatment, regardless of the location of the patient or the health care provider. Additionally, mHealth data are stored in servers, and consequently, data management that prevents all forms of manipulation is crucial for both medical practice and clinical trials.

**Objective:**

The aim of this study was to develop and evaluate a tamper-resistant mHealth system using blockchain technology, which enables trusted and auditable computing using a decentralized network.

**Methods:**

We developed an mHealth system for cognitive behavioral therapy for insomnia using a smartphone app. The volunteer data collected with the app were stored in JavaScript Object Notation format and sent to the blockchain network. Thereafter, we evaluated the tamper resistance of the data against the inconsistencies caused by artificial faults.

**Results:**

Electronic medical records collected using smartphones were successfully sent to a private Hyperledger Fabric blockchain network. We verified the data update process under conditions where all the validating peers were running normally. The mHealth data were successfully updated under network faults. We further ensured that any electronic health record registered to the blockchain network was resistant to tampering and revision. The mHealth data update was compatible with tamper resistance in the blockchain network.

**Conclusions:**

Blockchain serves as a tamperproof system for mHealth. Combining mHealth with blockchain technology may provide a novel solution that enables both accessibility and data transparency without a third party such as a contract research organization.

## Introduction

Digital health, including the utilization of mobile health (mHealth) apps and devices, has become popular in the everyday practice of medicine [1]. It has the potential to promote improved patient health outcomes, support care coordination, and improve communication. Whereas digital health has the potential for better patient care, there’s a need to consider the security issues [2]. Data tampering is one of the most crucial security risks [3]. If data tampering occurs during an attack on the system, it leads to a loss of data reliability. As data reliability is essential, especially for clinical trials, a tamperproof system is needed. Also, decision making in medical practice should be based on precise information from the patients.

Blockchain technology has attracted attention because of its efficacy in the prevention of data tampering. It serves as a distributed tamperproof database. To ensure tamper resistance, it maintains a continuously growing list of transactional records organized into blocks, using consensus algorithms that allow untrusted parties to agree on a common state. Valid transactions stored in a blockchain are digitally signed and timestamped by their sender, providing cryptographically irrefutable evidence of both the provenance and the existence of a record at a given time [4]. Bitcoin was the first implementation of blockchain as a digital asset in widespread use [5,6]. It is an electronic payment system based on cryptographic proof instead of trust. Although Bitcoin may be an appropriate technology for preventing data tampering in medical fields, it is currently not suitable for the following three reasons: (1) it is an open network that anyone can join; (2) it deals with currency, which is only one-dimensional data; and (3) it needs massive computing power to guarantee tamper resistance. However, a blockchain system that requires permission to join has been developed in a private network; this system could deal with multidimensional data, and it also does not need massive computing power for effective tamper resistance [7]. Beyond digital currency, researchers have started to focus on using blockchain methodology for building cryptographic proof of medical systems [8,9]. They have applied blockchain technology in the maintenance of protocols in clinical trials and for the management of electronic health records (EHRs) [4,10-14]. However, there has been no study to evaluate the use of blockchain technology in an mHealth system.

To address this issue, we have applied blockchain technology to an mHealth app that enables cognitive behavioral therapy for insomnia (CBTi) using a smartphone. Insomnia is a prevalent public health problem with a huge economic burden. Approximately 20% of the population meets the criteria for chronic insomnia as a disorder [15]. Insomnia is highly comorbid with various disorders such as hypertension [16], diabetes mellitus [17], and depression [18]. The combined direct and indirect economic burden associated with insufficient sleep is US $138 billion in Japan alone [19]. Given the high prevalence and detrimental effect of insomnia, effective and accessible treatment is crucial. CBTi is a first-line treatment with sufficient empirical support to be recommended for treating chronic insomnia [20]. It is a behavioral intervention that focuses on treating patients’ chronic insomnia through problem-solving techniques and supportive therapies to address some of the triggering factors [21]. Although there is plenty of evidence supporting the effectiveness of CBTi, the method is labor-intensive, expensive, and based at medical institutions. The lack of trained clinicians and high expenses limit access to CBTi and its dissemination. To overcome this obstacle, technological innovation has enabled delivery of CBTi using the Internet. Recent studies have shown that those who received Web-based CBTi had improved sleep outcomes [22-26]. In an mHealth system for CBTi, mobile devices and the host server are connected by a secured Internet network [25]. In the network, patients transfer their own EHRs from mobile devices, and data are stored in the server. Feedback advice based on the data is transferred to the patients’ mobile devices.

In this study, we developed an mHealth system for CBTi using a smartphone app together with blockchain storage platform and evaluated the tamper resistance of the data collected using smartphones.

## Methods

### The Structure of the mHealth System for CBTi

**Figure 1:**
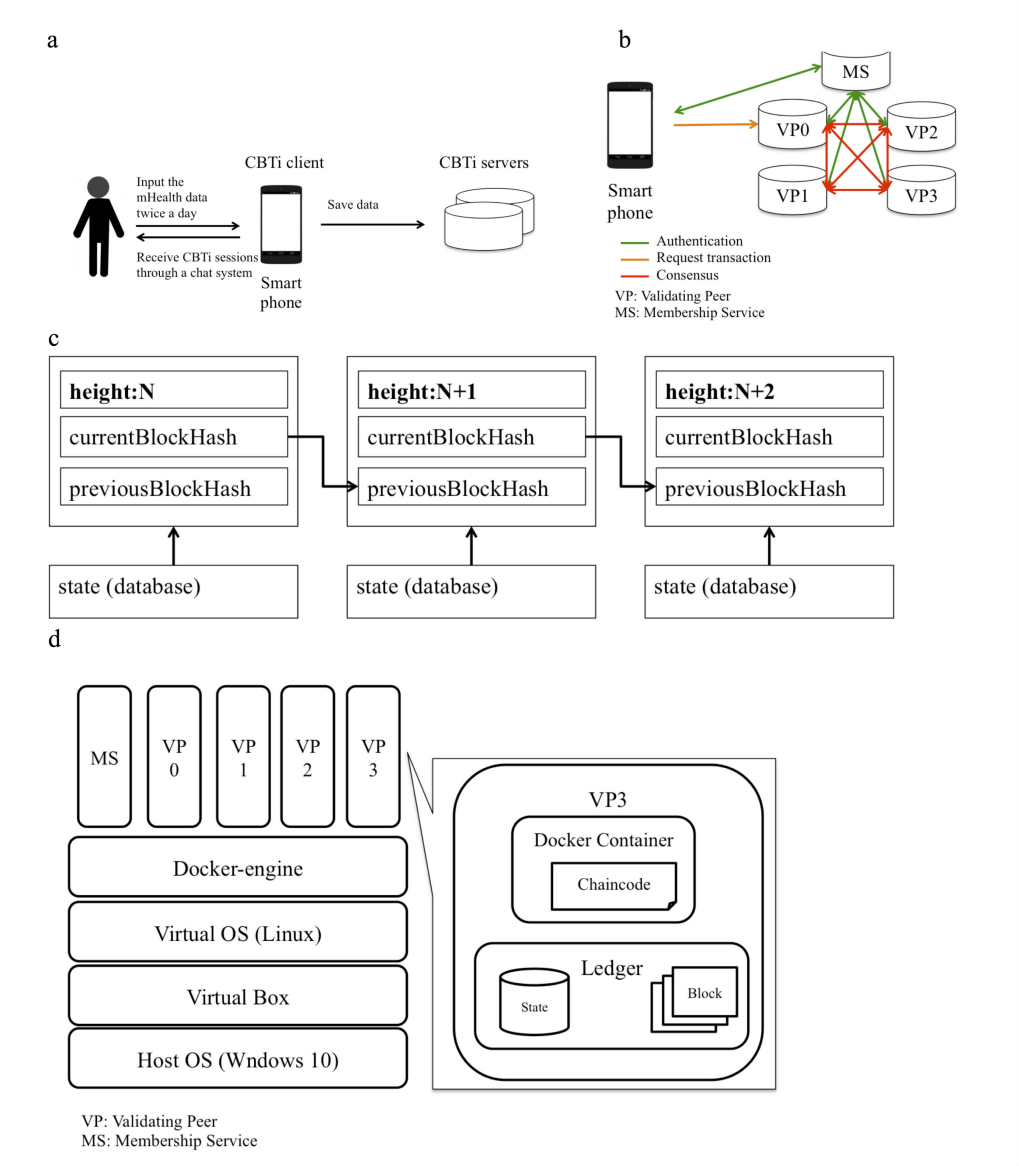
(a) The structure of the mobile health system for cognitive behavioral therapy for insomnia (b) The data update using a blockchain system (c) The structure of the blockchain (d) The virtual computing environment in the study.

Our mHealth system was composed of the CBTi client and the CBTi servers (Figure 1). In this system, patients received sessions through a chat program every day. The program comprised a fully automated smartphone app. Patients had to input their mHealth data twice a day, in the morning and in the evening. The CBTi sessions were conducted based on the collected data. The CBTi content covered not only behavioral and cognitive strategies but also relaxation strategies. The strategies were based on the current literature [21,27]. This system is used in our ongoing clinical trials (UMIN000023999).

### mHealth Records

The mHealth records collected from patients were divided into two types: subjective and objective data. The subjective data, which include clinical indicators, sleep status, and a review of daytime activities, were collected in the form of a self-administered questionnaire. The objective data, which include the results of a psychomotor vigilance test [28], were evaluated by measuring the touch response using the touch function of the smartphone. For the clinical indicators, the Athens Insomnia Scale [29], the Epworth Sleepiness Scale [30], and the Quick Inventory of Depressive Symptomatology were used [31]. For the sleep status, the time of going to bed, time of falling asleep, time of waking up, and time of getting up were recorded. All data were stored in the JavaScript Object Notation (JSON) format in the database. We utilized the mHealth data of a volunteer. Informed consent was obtained from the volunteer for publication of this study. The study has received ethical approval from the Institute of Neuropsychiatry Ethics Committee. All the methods were performed in accordance with the relevant guidelines and regulations.

### mHealth Data Registration to the Blockchain Network　

We show the data update process using the blockchain network (Figure 1). In the system, the patients send their own daily data via smartphones and get feedback information for the data. The system was constructed using smartphones and the cluster of servers on the network. We utilized Hyperledger Fabric version 0.5 to operate the system because Hyperledger is an open-source blockchain platform and has become widely used [7,32]. In this study, the system comprised 4 validating peers (VP) and a membership service (MS). A VP was in charge of the main function of the blockchain, and an MS was in charge of authentication for the client (smartphone) and the VPs. The MS issued enrollment and transaction certificates to the client, and the client used the certificates for the authentication. Every VP had a replica of the common database that was called the “state.”

One of the VPs became a leader of the network and accepted requests from the CBTi client. The request that was accepted by the leader was delivered to each VP. The CBTi client sent the first transaction to the leader VP, and then the leader VP let each VP install chaincode and perform the initialization. After that, the CBTi client sent the request for data processing, and the leader VP sent the request from the client to each VP. The VPs executed an installed chaincode and returned hash values generated from the execution result. At that time, each VP followed the consensus algorithm, which was called the Practical Byzantine Fault Tolerance (PBFT) algorithm [33,34]. When the VPs reached a consensus, it was settled among all VPs. Thereafter, each VP stored the same result into their state. After that, the information based on the hashed result of the transaction was generated. This was called the “block.” That block contained the previous block information as a hash value and the current block hash value (Figure 1). Figure 1 illustrates the structure of the blockchain. The field “height” was the length of the blockchain (N: positive integer). At the start of each process, height was 1, and it increased incrementally with the generation of the blocks. Each block, except the initial block, includes 3 fields: “currentBlockHash,” “previousBlockHash,” and “statehash.” The field “currentBlockHash” was the current hash information of the block and matched “previousBlockHash” of the next block. The block also preserved the hashed information of the current state. The new block generated in this way was connected to the list, which is called the “blockchain.”

### Test Scenario

We evaluated the network robustness of the CBTi system with regard to data integrity according to the Recommendation of the Council Concerning Guidelines for the Security of Information Systems and Networks [35].

To test network robustness during a network fault, we ensured the correctness of mHealth data updates from a smartphone using the procedure described below. First, we verified the process of normal data update. Next, we tested the data updates when one of the VP servers was down.

For the test, we utilized the mHealth data of a volunteer over the course of 5 days. The client data format was JSON and, in the experiments, the data were input manually to the CBTi servers instead of via the smartphone app. Each server was constructed in the virtual environment, which ran in the same local personal computer with Intel Core i5-5200U CPU 2.2GHz and 8GB memory running Windows 10. For the construction of the virtual environment, we utilized Docker version 1.10.2 [36], Oracle VirtualBox version 5.1.12, and Vagrant version 1.9.1. We used docker-compose version 1.5.2 to manage Docker. The virtual computing environment in the study comprised 4 VPs and an MS (Figure 1). Each VP server comprised a Docker container and a ledger. A chaincode was registered in the docker container. The ledger comprised state and blocks. The state was the key-value store database and recorded the result of the transactions.

## Results

### Normal Data Update

We verified the data update process under conditions where all the VPs were running normally. The test procedure was divided into 2 steps: Deploy and Invoke.

#### The Deploy Step Execution

We started the CBTi servers composed of 4 VPs and an MS. We initialized the state with the user data for 2 days and deployed the chaincode on each of the VPs. This is the Deploy step. In detail, the steps were as follows: First, we logged into the CBTi system with a user ID and password. We then initialized the state using the user data of a nonpatient volunteer for 2 days. Next, we deployed a chaincode to each of the 4 VPs. The chaincode describes the procedure for the addition of JSON formed data to the database. When the Deploy step was executed successfully, the block based on the transaction information was produced, and user data were added to the state.

**Figure 2:**
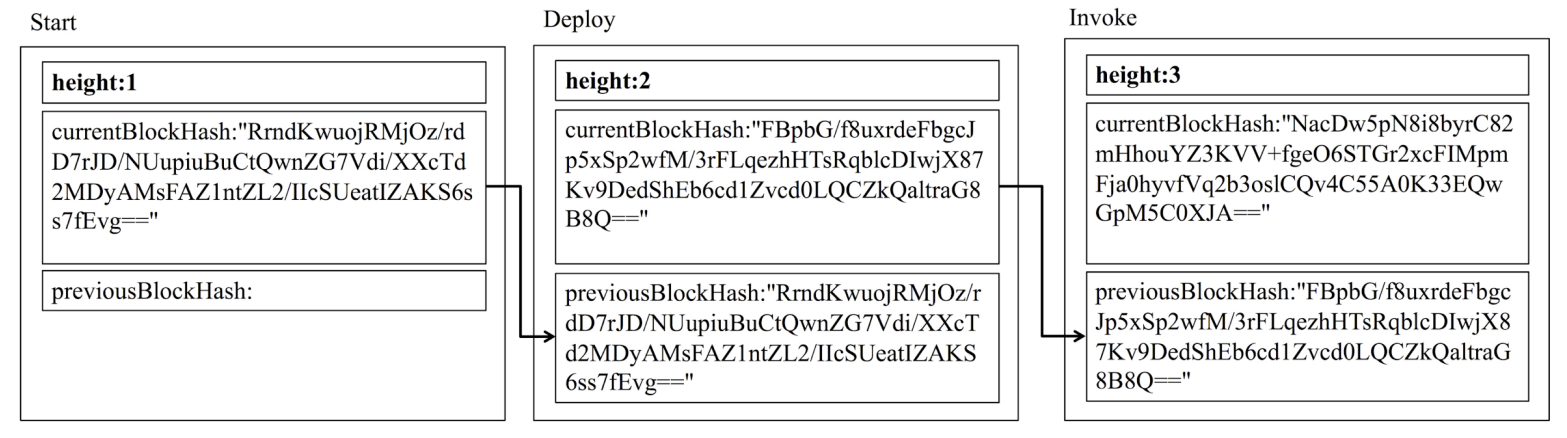
The blockchain (excerpt) in the normal mobile health data update.

**Figure 3:**
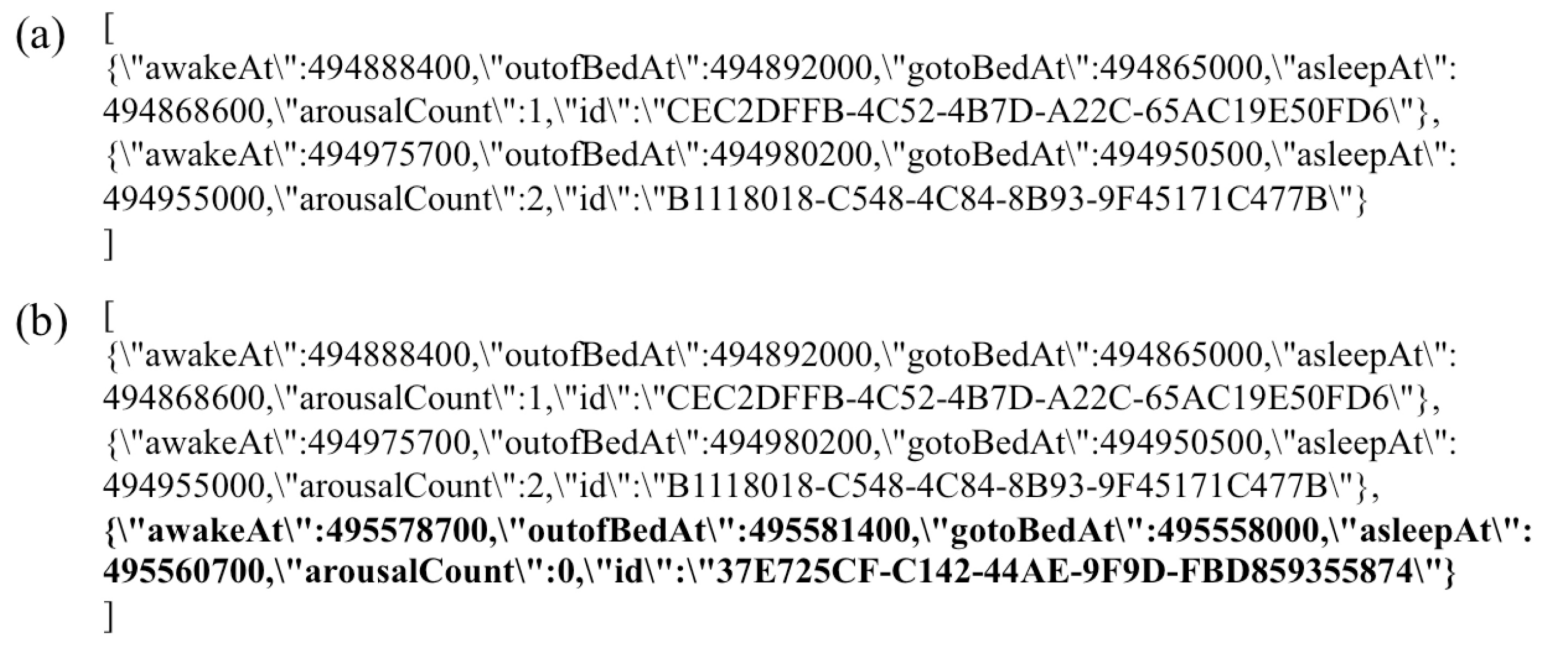
The user data (excerpt) queried from the state in the normal mobile health data update. (a) The initial user data after the Deploy step. (b) The updated user data after the Invoke step (newly added data were highlighted).

We ensured that the block was generated successfully and that the height (the length of the blockchain) incremented from the one at the start of the normal data update (Figure 2). At the start of the normal data update, the height was 1 and incremented with the production of the blocks. The “currentBlockHash” field matched the “previousBlockHash” field of the next block. At the start of the normal data update, the “previousBlockHash” field had no data. The queried user data from the state showed the user data for 2 days (Figure 3). The user data for 2 days were registered to the state as the initial data. Thus, the user data were registered to the state successfully.

#### The Invoke Step Execution

We executed the transaction to update the database with user data for a day using the chaincode on each VP. This is called the Invoke step. We ran the deployed chaincode on each VP, and each chaincode produced a temporary result. When the VPs in the network reached a consensus based on hash information of the temporary results, the transaction was confirmed. When the transaction was confirmed successfully, the user data were updated to the state, after which the block was produced. We ensured the production of the block and the increment of the height from one of the Deploy step (Figure 2). At the start of the normal data update, the height was 1 and incremented with the production of the blocks. The “currentBlockHash” field matched the “previousBlockHash” field of the next block. At the start of the normal data update, the “previousBlockHash” field had no data.

We further confirmed the success of the data update by querying it. We could see that user data for a day had been added. The excerpt of user data registered to the database is shown in Figure 3. The user data for each day were added to the state. The full information from the produced blockchain and user data in the normal data update is shown in Multimedia Appendix 1. Taken together, we could register and update the EHRs that were recorded from the smartphone into the blockchain network.

### Validation of Tamper Resistance

**Figure 4:**
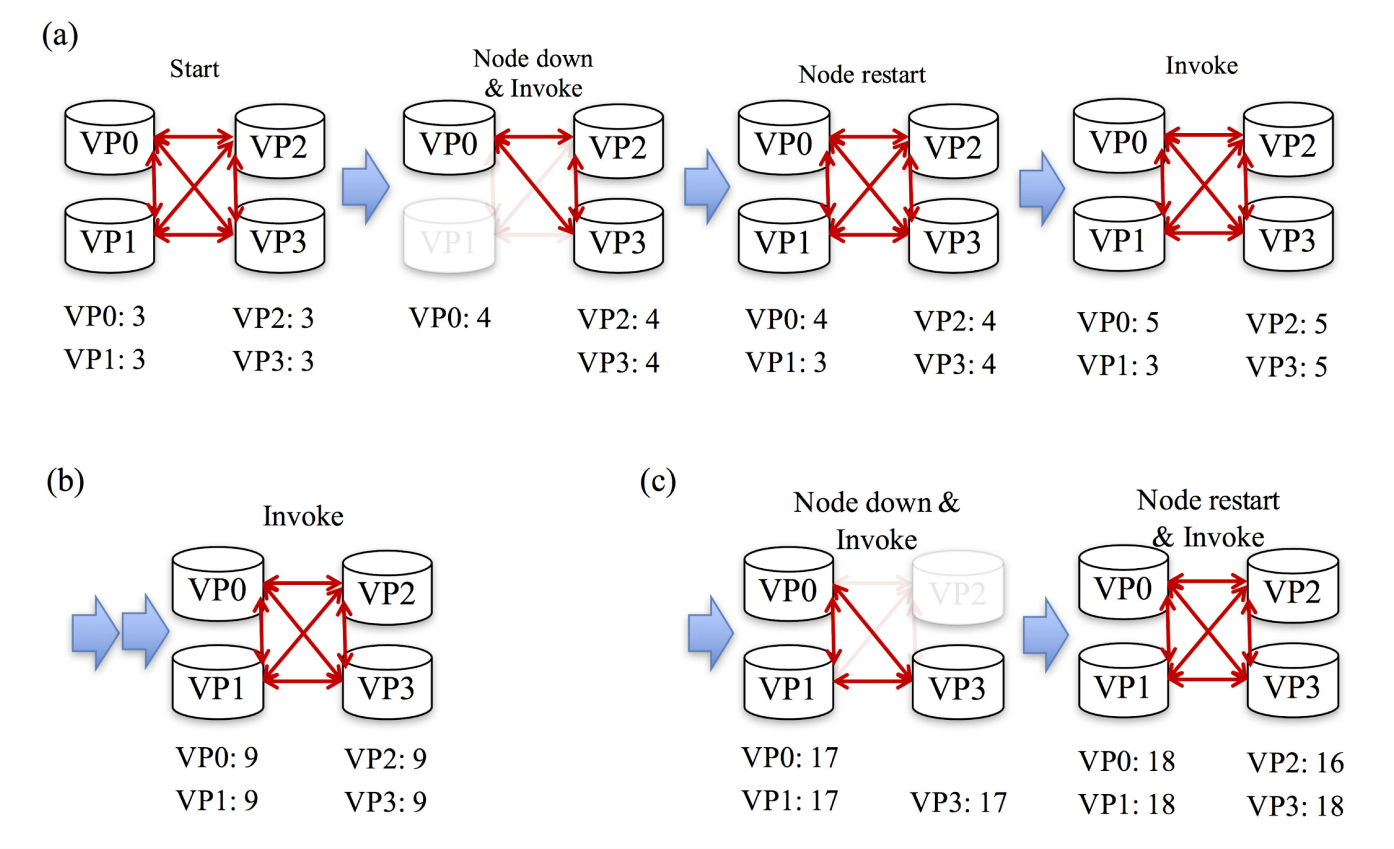
The blockchain in the mobile health data update test when one of validating peers (VPs) was down. The blockchain height of each VP is shown. (a) Robustness of the blockchain network against network failure. (b) Correction of the inconsistency by ledger synchronization. (c) Rejoining the Practical Byzantine Fault Tolerance consensus after another network failure.

**Figure 5:**
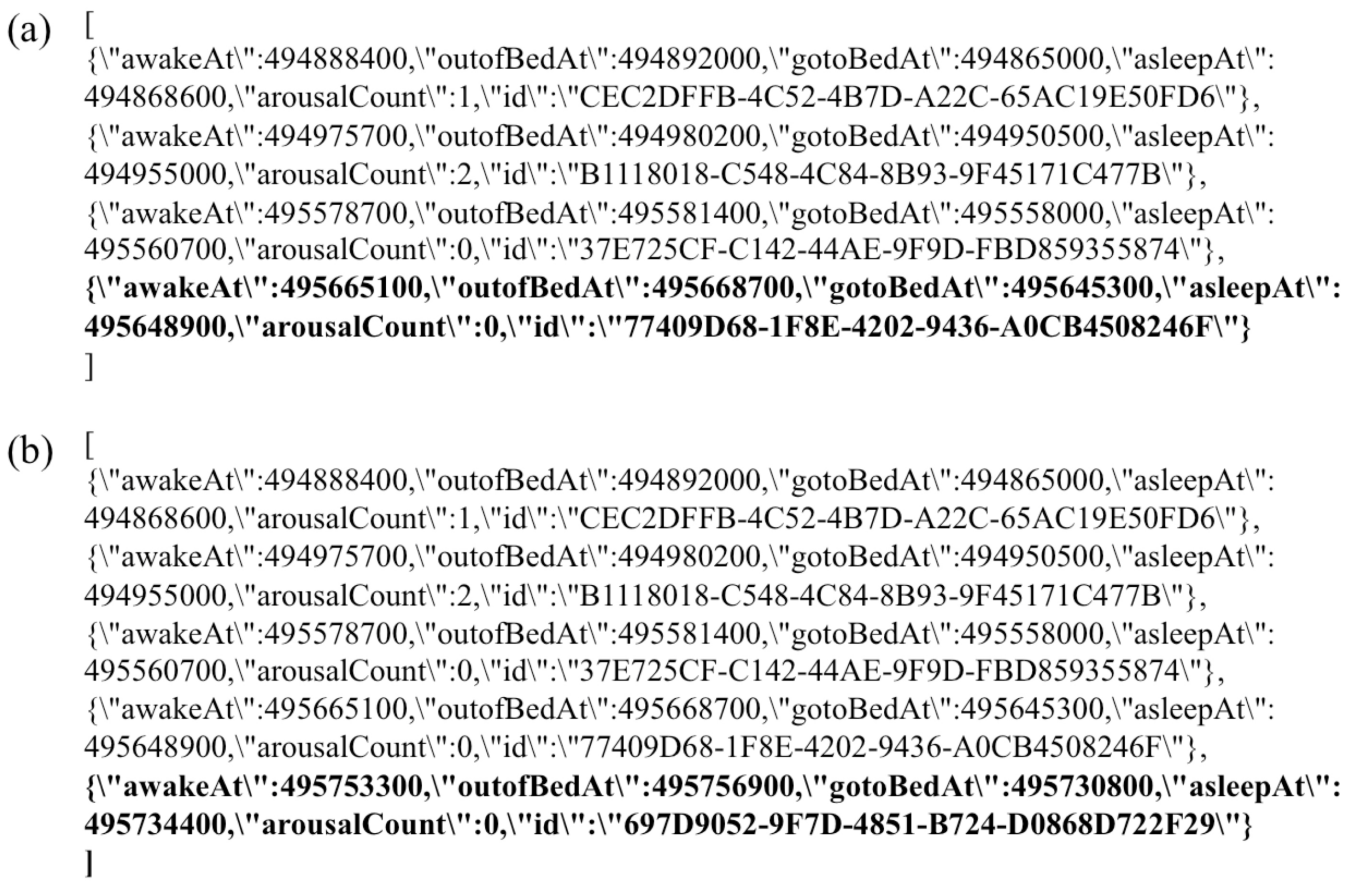
The user mobile health data (excerpt) queried from the state in the data update test when one of the validating peers (VPs) was down. (a) The successfully added user data after the Invoke step when VP1 was down (newly added data were highlighted). (b) The user data after the Invoke step when VP1 was rebooted (newly added data were highlighted).

To investigate the tamper resistance of our system, we produced an artificial fault in the system that caused ledgers in the VPs to contain inconsistencies. We produced a network fault by taking one of the VPs down and then updated data during the network fault. This gave an indication of the robustness of the blockchain network. After rebooting the VP that had stopped, we confirmed that the data in the rebooted VP were one step behind. We also checked that the inconsistency was corrected by ledger synchronization. In detail, the process was as follows: First, there were 4 VPs running in the initial state. We tested sequentially after normal data updates. Thus, the user data for 3 days was recorded (Figure 3). Second, we stopped one of the VPs (VP1); therefore, the total remaining number of running VPs was 3 (VP0, VP2, VP3). We then executed the Invoke step. Using PBFT as a consensus protocol, a blockchain network of N nodes can withstand a number of failed nodes, f, where f=(N−1)/3. Our network contains N=4 nodes, so applying the formula for the maximum number of tolerated failed nodes results in f=(4−1)/3=1. In other words, PBFT ensures that a minimum of 2×f + 1 (that is 3) nodes reach consensus on the order of transactions before appending them to the shared ledger. The block was produced (Figure 4: Node down & Invoke), and the state was updated successfully because of the PBFT consensus protocol (Figure 5). At the start of all of the processes in the data update test, the height was 3 and increased incrementally with the production of the blocks. This suggests that the mHealth system with the blockchain network is robust against network faults.

Next, we rebooted the stopped VP (VP1). We confirmed that the block of VP1 was one step behind because VP1 had been down (Figure 4: Node restart). As of this point, the total number of running VPs was 4. We executed the Invoke step again. The block was produced successfully (Figure 4: Invoke), and the state was updated (Figure 5).

The full user data queried from the state are shown in Multimedia Appendix 2. Because only a minimum of 2×f + 1 nodes must reach consensus before proceeding to the next block of transactions, the ledger on any additional nodes (beyond 2×f + 1) will temporarily lag behind. The node that was restarted tries to synchronize with the latest ledger after several transactions (Figure 4).

We further tested whether the rebooted VP (VP1) could rejoin the PBFT consensus if another VP (VP2) was temporarily down. After VP2 was offline, VP1 completely caught up with VP0 and VP3 because 2×f + 1 nodes must reach consensus before proceeding to the next block of transactions (Figure 4).

The full information for the blockchain from these experiments is shown in Multimedia Appendix 3. These results indicate that the EHR registered to the blockchain network is resistant to tampering and revision. The update of mHealth data was also compatible with tamper resistance in the blockchain network.

## Discussion

### Principal Findings

In this study, we have developed and evaluated a tamper-resistant mHealth system using the blockchain technique. The mHealth data collected using a smartphone were sent to a private Hyperledger Fabric blockchain network. The mHealth database in the blockchain network was robust against network faults such as “node down.” The node of the distributed database in the blockchain network that was down could catch up with other normal nodes because of the consensus algorithm, which is not implemented in ordinary distributed database systems. Therefore, the distributed database in the blockchain network was resistant to tampering and revision, and the mHealth data update was compatible with tamper resistance in the blockchain network.

Thus, mHealth technologies such as CBTi using a mobile device enable delivery of treatments that have previously been labor-intensive. The mHealth system needs to be tamper-resistant because the system automatically provides treatment to patients based on the stored data. Recently, attacks to hospital networks using ransomware have been reported where hospitals had to pay ransom to the attackers [37]. If an mHealth system is attacked and the data is tampered with, the feedback based on the tampered information may be harmful to the patients.

In previous studies, various secure EHR systems have been proposed [14,38,39]. It has been pointed out that such systems are inadequate for practical use as they have security risks because of their reliance on a single trusted authority [40]. This study avoided this risk by using a distributed blockchain network. Moreover, the system we constructed in this study utilized open-source software that could be applied to other mHealth systems.

There are two reasons that blockchain technology is favorable to mHealth data. First, as shown in this study, the mHealth data update was not frequent because the patients’ data were transferred to the server only twice a day in our system. So, although blockchain is not ideal for data with high temporal resolution, it could easily deal with mHealth data. Second, mHealth data are valuable, which is why a high level of security is essential. From the point of the view of security, blockchain is expected to accomplish high tamper resistance.

The system guarantees the accuracy of mHealth data without confirmation by a third party, so it has the potential for use in clinical trials in the following two ways: (1) the system would reduce the cost in clinical trials by decreasing the amount currently spent on confirmation by a third party such as a contract research organization [41]; and (2) it could reduce the possibility of human error because the system could minimize human involvement with the data. Furthermore, one of the ethical problems in clinical trials is that patient data and personal information can be accessed by people who are not directly involved in that patient’s care. Thus, the use of blockchain technology in clinical trials may enhance the development of drugs and medical devices.

### Limitations

This study has two limitations. First, there is vulnerability around the blockchain system. Although blockchain technology is tamper-resistant, the implementation around it can be attacked. Poorly maintained and outdated codes allowed vulnerability in an incident involving a decentralized autonomous organization [42]. Second, the theoretical limitation of the consensus algorithm used in the blockchain also has vulnerability. Although we utilized the PBFT algorithm for the consensus, the blockchain can be disabled if more than (N-1)/3 of the VPs are attacked at the same time. Such incidents could happen, especially in small networks [43]. To solve this problem, it is important to increase the number of servers, and at the same time, increase the number of stakeholders holding the servers to prevent malicious users from occupying the system. At the moment, private blockchain can scale to a few hundred nodes, and an advanced system has been developed [44].

### Conclusions

In this study, we developed and evaluated a tamper-resistant mobile health care system using blockchain technology.

## Appendix

Multimedia Appendix 1 The user data queried from the state in the normal data update.

Multimedia Appendix 2 The user data queried from the state in the data update test when one of VPs was down.

Multimedia Appendix 3 The blockchain information in the validation test of tamper resistance.

## Abbreviations

CBTi: cognitive behavioral therapy for insomnia
EHRs: electronic health records
JSON: JavaScript Object Notation
mHealth: mobile health
MS: membership service
PBFT: Practical Byzantine Fault Tolerance
VP: validating peer
