# Supplementary material for: Tamper-Resistant Mobile Health Using Blockchain Technology
Source: JMIR Mhealth Uhealth. 2017 Jul 26;5(7):e111. doi: 10.2196/mhealth.7938 (PMC5550736; doi:10.2196/mhealth.7938)
Supplement: Multimedia Appendix 2 [file mhealth_v5i7e111_app2.pdf]

Supplemental Data 2 The user data queried from the state in the data update test when one of VPs was down

(a) The user data after the Invoke step when VP1 was down. (b) The user data after the Invoke step when VP1 was rebooted. The excerpt user data are shown in Figure 5.

(a)

{"jsonrpc":"2.0","result":{"status":"OK","message":"{\"sleeps\":[{\"awakeAt\":494888400,\"outofBedAt\":
494892000,\"gotoBedAt\":494865000,\"asleepAt\":494868600,\"arousalCount\":1,\"id\":
\"CEC2DFFB-4C52-4B7D-A22C-65AC19E50FD6\"},{\"awakeAt\":494975700,\"outofBedAt\":
494980200,\"gotoBedAt\":494950500,\"asleepAt\":494955000,\"arousalCount\":2,\"id\":\"B1118018-
C548-4C84-8B93-9F45171C477B\"},{\"awakeAt\":495578700,\"outofBedAt\":495581400,\"gotoBedAt\":
495558000,\"asleepAt\":495560700,\"arousalCount\":0,\"id\":\"37E725CF-C142-44AE-9F9D-
FBD859355874\"},{\"awakeAt\":495665100,\"outofBedAt\":495668700,\"gotoBedAt\":495645300,\"asleepAt
\":495648900,\"arousalCount\":0,\"id\":\"77409D68-1F8E-4202-9436-A0CB4508246F\"}],\"esses\":
[{\"answers\":[2,2,2,2,2,2,2,2],\"date\":494939381},{\"answers\":[2,2,2,3,2,0,2,0],\"date\":495634532},
{\"answers\":[2,2,1,3,2,0,2,0],\"date\":496484154}],\"days\":[{\"mistakeCount\":0,\"ess\":{\"answers\":
[2,2,2,2,2,2,2,2],\"date\":494939381},\"reviewedEvening\":true,\"day_activity_check_items\":0,\"msec\":
0,\"activities\":[{\"id\":0,\"title\":\"Sleep efficiency\"},{\"id\":0,\"title\":\"Sleep efficiency\"}],\"date\":
494938618,\"reviewedMorning\":false,\"timestamp\":494938618,\"lapseCount\":0,\"depression\":{\"answers\":
[1,1,1,0,0,1,0,1,0,1,1,0,0,1,0,0],\"date\":494938903},\"noSleep\":false},{\"mistakeCount\":0,\"ess\":{\"answers
\":[0,0,0,0,0,0,0,0],\"date\":0},\"reviewedEvening\":false,\"day_activity_check_items\":0,\"msec\":0,\"activities
\":null,\"date\":494852218,\"reviewedMorning\":false,\"timestamp\":494852218,\"lapseCount\":0,\"depression
\":{\"answers\":[0,0,0,0,0,0,0,0,0,0,0,0,0,0,0,0],\"date\":0},\"noSleep\":false},{\"mistakeCount\":0,\"ess\":
{\"answers\":[0,0,0,0,0,0,0,0],\"date\":0},\"reviewedEvening\":false,\"day_activity_check_items\":0,\"msec\":
0,\"activities\":null,\"date\":494765818,\"reviewedMorning\":false,\"timestamp\":494765818,\"lapseCount\":
0,\"depression\":{\"answers\":[0,0,0,0,0,0,0,0,0,0,0,0,0,0,0,0],\"date\":0},\"noSleep\":false},{\"mistakeCount\":
0,\"ess\":{\"answers\":[0,0,0,0,0,0,0,0],\"date\":0},\"reviewedEvening\":false,\"day_activity_check_items\":
0,\"msec\":0,\"activities\":null,\"date\":494679418,\"reviewedMorning\":false,\"timestamp\":
494679418,\"lapseCount\":0,\"depression\":{\"answers\":[0,0,0,0,0,0,0,0,0,0,0,0,0,0,0,0],\"date\":0},\"noSleep
\":false},{\"mistakeCount\":0,\"ess\":{\"answers\":[0,0,0,0,0,0,0,0],\"date\":0},\"reviewedEvening\":false,
\"day_activity_check_items\":0,\"msec\":0,\"activities\":null,\"date\":494593018,\"reviewedMorning\":false,
\"timestamp\":494593018,\"lapseCount\":0,\"depression\":{\"answers\":[0,0,0,0,0,0,0,0,0,0,0,0,0,0,0,0],\"date\":
0},\"noSleep\":false},{\"mistakeCount\":0,\"ess\":{\"answers\":[0,0,0,0,0,0,0,0],\"date\":0},\"reviewedEvening
\":false,\"day_activity_check_items\":0,\"msec\":0,\"activities\":null,\"date\":494506618,\"reviewedMorning
\":false,\"timestamp\":494506618,\"lapseCount\":0,\"depression\":{\"answers\":[0,0,0,0,0,0,0,0,0,0,0,0,0,0,0,0],
\"date\":0},\"noSleep\":false},{\"mistakeCount\":0,\"ess\":{\"answers\":[0,0,0,0,0,0,0,0],\"date\":0},
\"reviewedEvening\":false,\"day_activity_check_items\":0,\"msec\":0,\"activities\":null,\"date\":
494420218,\"reviewedMorning\":false,\"timestamp\":494420218,\"lapseCount\":0,\"depression\":{\"answers\":
[0,0,0,0,0,0,0,0,0,0,0,0,0,0,0,0],\"date\":0},\"noSleep\":false},{\"mistakeCount\":0,\"ess\":{\"answers\":
[0,0,0,0,0,0,0,0],\"date\":0},\"reviewedEvening\":false,\"day_activity_check_items\":0,\"msec\":0,\"activities
\":null,\"date\":494333818,\"reviewedMorning\":false,\"timestamp\":494333818,\"lapseCount\":0,\"depression
\":{\"answers\":[0,0,0,0,0,0,0,0,0,0,0,0,0,0,0,0],\"date\":0},\"noSleep\":false},{\"mistakeCount\":0,\"ess\":
{\"answers\":[2,2,2,3,2,0,2,0],\"date\":495634532},\"reviewedEvening\":true,\"day_activity_check_items\":
0,\"msec\":492,\"activities\":[{\"id\":0,\"title\":\"Reviewing of sleep\"},{\"id\":0,\"title\":\"Reviewing of sleep
\"},{\"id\":0,\"title\":\"Recording of sleep\"},{\"id\":0,\"title\":\"Sleep efficiency\"},{\"id\":0,\"title\":
\"Sleepiness in the daytime\"},{\"id\":0,\"title\":\"Cognitive therapy\"}],\"date\":495605869,\"reviewedMorning
\":false,\"timestamp\":495605869,\"lapseCount\":7,\"depression\":{\"answers\":[0,0,0,0,0,0,0,0,0,0,0,0,0,0,0,0],
\"date\":0},\"noSleep\":false}],\"build\":{\"build_version_80\":494939250,\"build_version_83\":
495771269,\"build_version_84\":496221360},\"goals\":[{\"arouseMinute\":0,\"arouseHour\":7,\"sleepHour\":
23,\"sleepMinute\":0,\"startOn\":493210869}],\"aises\":[],\"depressions\":[{\"answers\":
[1,1,1,0,0,1,0,1,0,1,1,0,0,1,0,0],\"date\":494938903}],\"activities\":[{\"id\":0,\"title\":\"Sleep efficiency\"},{\"id
\":0,\"title\":\"Sleep efficiency\"},{\"id\":0,\"title\":\"Reviewing of sleep\"},{\"id\":0,\"title\":\"Reviewing of
sleep\"},{\"id\":0,\"title\":\"Recording of sleep\"},{\"id\":0,\"title\":\"Sleep efficiency\"},{\"id\":0,\"title\":
\"Sleepiness in the daytime\"},{\"id\":0,\"title\":\"Cognitive therapy\"},{\"id\":0,\"title\":\"Reviewing of sleep
\"}],\"timeInfo\":{\"createdAt\":0,\"updatedAt\":0},\"user\":{\"startedOn\":494938701,\"gender\":2,\"id\":
\"DS0010-1\",\"medicineDescription\":\"Abc\",\"stature\":160,\"age\":25,\"weight\":50,\"usingMedicine\":true,
\"actigraphId\":\"0123456\",\"initialAisScore\":10}}"},"id":5}

(b)

{"jsonrpc":"2.0","result":{"status":"OK","message":"{\"sleeps\":[{\"awakeAt\":494888400,\"outofBedAt\":
494892000,\"gotoBedAt\":494865000,\"asleepAt\":494868600,\"arousalCount\":1,\"id\":
\"CEC2DFFB-4C52-4B7D-A22C-65AC19E50FD6\"},{\"awakeAt\":494975700,\"outofBedAt\":
494980200,\"gotoBedAt\":494950500,\"asleepAt\":494955000,\"arousalCount\":2,\"id\":\"B1118018-
C548-4C84-8B93-9F45171C477B\"},{\"awakeAt\":495578700,\"outofBedAt\":495581400,\"gotoBedAt\":
495558000,\"asleepAt\":495560700,\"arousalCount\":0,\"id\":\"37E725CF-C142-44AE-9F9D-
FBD859355874\"},{\"awakeAt\":495665100,\"outofBedAt\":495668700,\"gotoBedAt\":495645300,\"asleepAt
\":495648900,\"arousalCount\":0,\"id\":\"77409D68-1F8E-4202-9436-A0CB4508246F\"},{\"awakeAt\":
495753300,\"outofBedAt\":495756900,\"gotoBedAt\":495730800,\"asleepAt\":495734400,\"arousalCount\":
0,\"id\":\"697D9052-9F7D-4851-B724-D0868D722F29\"}],\"esses\":[{\"answers\":[2,2,2,2,2,2,2,2],\"date\":
494939381},{\"answers\":[2,2,2,3,2,0,2,0],\"date\":495634532},{\"answers\":[2,2,1,3,2,0,2,0],\"date\":
496484154},{\"answers\":[1,2,1,3,2,0,2,0],\"date\":496656633},{\"answers\":[1,1,1,3,2,1,1,0],\"date\":
497527246}],\"days\":[{\"mistakeCount\":0,\"ess\":{\"answers\":[2,2,2,2,2,2,2,2],\"date\":494939381},
\"reviewedEvening\":true,\"day_activity_check_items\":0,\"msec\":0,\"activities\":[{\"id\":0,\"title\":\"Sleep
efficiency\"},{\"id\":0,\"title\":\"Sleep efficiency\"}],\"date\":494938618,\"reviewedMorning\":false,
\"timestamp\":494938618,\"lapseCount\":0,\"depression\":{\"answers\":[1,1,1,0,0,1,0,1,0,1,1,0,0,1,0,0],\"date\":
494938903},\"noSleep\":false},{\"mistakeCount\":0,\"ess\":{\"answers\":[0,0,0,0,0,0,0,0],\"date\":0},
\"reviewedEvening\":false,\"day_activity_check_items\":0,\"msec\":0,\"activities\":null,\"date\":
494852218,\"reviewedMorning\":false,\"timestamp\":494852218,\"lapseCount\":0,\"depression\":{\"answers\":
[0,0,0,0,0,0,0,0,0,0,0,0,0,0,0,0],\"date\":0},\"noSleep\":false},{\"mistakeCount\":0,\"ess\":{\"answers\":
[0,0,0,0,0,0,0,0],\"date\":0},\"reviewedEvening\":false,\"day_activity_check_items\":0,\"msec\":0,\"activities
\":null,\"date\":494765818,\"reviewedMorning\":false,\"timestamp\":494765818,\"lapseCount\":0,\"depression
\":{\"answers\":[0,0,0,0,0,0,0,0,0,0,0,0,0,0,0,0],\"date\":0},\"noSleep\":false},{\"mistakeCount\":0,\"ess\":
{\"answers\":[0,0,0,0,0,0,0,0],\"date\":0},\"reviewedEvening\":false,\"day_activity_check_items\":0,\"msec\":
0,\"activities\":null,\"date\":494679418,\"reviewedMorning\":false,\"timestamp\":494679418,\"lapseCount\":
0,\"depression\":{\"answers\":[0,0,0,0,0,0,0,0,0,0,0,0,0,0,0,0],\"date\":0},\"noSleep\":false},{\"mistakeCount\":
0,\"ess\":{\"answers\":[0,0,0,0,0,0,0,0],\"date\":0},\"reviewedEvening\":false,\"day_activity_check_items\":
0,\"msec\":0,\"activities\":null,\"date\":494593018,\"reviewedMorning\":false,\"timestamp\":
494593018,\"lapseCount\":0,\"depression\":{\"answers\":[0,0,0,0,0,0,0,0,0,0,0,0,0,0,0,0],\"date\":0},\"noSleep
\":false},{\"mistakeCount\":0,\"ess\":{\"answers\":[0,0,0,0,0,0,0,0],\"date\":0},\"reviewedEvening\":false,
\"day_activity_check_items\":0,\"msec\":0,\"activities\":null,\"date\":494506618,\"reviewedMorning\":false,
\"timestamp\":494506618,\"lapseCount\":0,\"depression\":{\"answers\":[0,0,0,0,0,0,0,0,0,0,0,0,0,0,0,0],\"date\":
0},\"noSleep\":false},{\"mistakeCount\":0,\"ess\":{\"answers\":[0,0,0,0,0,0,0,0],\"date\":0},\"reviewedEvening
\":false,\"day_activity_check_items\":0,\"msec\":0,\"activities\":null,\"date\":494420218,\"reviewedMorning
\":false,\"timestamp\":494420218,\"lapseCount\":0,\"depression\":{\"answers\":[0,0,0,0,0,0,0,0,0,0,0,0,0,0,0,0],
\"date\":0},\"noSleep\":false},{\"mistakeCount\":0,\"ess\":{\"answers\":[0,0,0,0,0,0,0,0],\"date\":0},
\"reviewedEvening\":false,\"day_activity_check_items\":0,\"msec\":0,\"activities\":null,\"date\":
494333818,\"reviewedMorning\":false,\"timestamp\":494333818,\"lapseCount\":0,\"depression\":{\"answers\":
[0,0,0,0,0,0,0,0,0,0,0,0,0,0,0,0],\"date\":0},\"noSleep\":false},{\"mistakeCount\":0,\"ess\":{\"answers\":
[2,2,2,3,2,0,2,0],\"date\":495634532},\"reviewedEvening\":true,\"day_activity_check_items\":0,\"msec\":
492,\"activities\":[{\"id\":0,\"title\":\"Reviewing of sleep\"},{\"id\":0,\"title\":\"Reviewing of sleep\"},{\"id\":
0,\"title\":\"Recording of sleep\"},{\"id\":0,\"title\":\"Sleep efficiency\"},{\"id\":0,\"title\":\"Sleepiness in the
daytime\"},{\"id\":0,\"title\":\"Cognitive therapy\"}],\"date\":495605869,\"reviewedMorning\":false,
\"timestamp\":495605869,\"lapseCount\":7,\"depression\":{\"answers\":[0,0,0,0,0,0,0,0,0,0,0,0,0,0,0,0],\"date\":
0},\"noSleep\":false},{\"mistakeCount\":0,\"ess\":{\"answers\":[0,0,0,0,0,0,0,0],\"date\":0},\"reviewedEvening
\":false,\"day_activity_check_items\":29167,\"msec\":0,\"activities\":[{\"id\":0,\"title\":\"Reviewing of sleep\"},
{\"id\":0,\"title\":\"Reviewing of sleep\"},{\"id\":0,\"title\":\"Reviewing of sleep\"}],\"date\":
495680001,\"reviewedMorning\":true,\"timestamp\":495680001,\"lapseCount\":0,\"depression\":{\"answers\":
[0,0,0,0,0,0,0,0,0,0,0,0,0,0,0,0],\"date\":0},\"noSleep\":false}],\"build\":{\"build_version_80\":
0,\"build_version_83\":0,\"build_version_84\":0},\"goals\":null,\"aises\":[],\"depressions\":[{\"answers\":
[1,1,1,0,0,1,0,1,0,1,1,0,0,1,0,0],\"date\":494938903}],\"activities\":[{\"id\":0,\"title\":\"Sleep efficiency\"},{\"id
\":0,\"title\":\"Sleep efficiency\"},{\"id\":0,\"title\":\"Reviewing of sleep\"},{\"id\":0,\"title\":\"Reviewing of
sleep\"},{\"id\":0,\"title\":\"Recording of sleep\"},{\"id\":0,\"title\":\"Sleep efficiency\"},{\"id\":0,\"title\":
\"Sleepiness in the daytime\"},{\"id\":0,\"title\":\"Cognitive therapy\"},{\"id\":0,\"title\":\"Reviewing of sleep
\"},{\"id\":0,\"title\":\"Reviewing of sleep\"},{\"id\":0,\"title\":\"Reviewing of sleep\"},{\"id\":0,\"title\":
