# Supplementary material for: Tamper-Resistant Mobile Health Using Blockchain Technology
Source: JMIR Mhealth Uhealth. 2017 Jul 26;5(7):e111. doi: 10.2196/mhealth.7938 (PMC5550736; doi:10.2196/mhealth.7938)
Supplement: Multimedia Appendix 3 [file mhealth_v5i7e111_app3.pdf]

Supplemental Data 3 The blockchain information in the experiments shown in Figure 4

]$ ./get_block.sh

vp0

{"height":1,"currentBlockHash":"RrndKwuojRMjOz/rdD7rJD/NUupiuBuCtQwnZG7Vdi/XXcTd2MDyAMsFAZ1ntZL2/IlcSUeatIZAKS6ss7fEvg=="}

vp1

{"height":1,"currentBlockHash":"RrndKwuojRMjOz/rdD7rJD/NUupiuBuCtQwnZG7Vdi/XXcTd2MDyAMsFAZ1ntZL2/IlcSUeatIZAKS6ss7fEvg=="}

vp2

{"height":1,"currentBlockHash":"RrndKwuojRMjOz/rdD7rJD/NUupiuBuCtQwnZG7Vdi/XXcTd2MDyAMsFAZ1ntZL2/IlcSUeatIZAKS6ss7fEvg=="}

vp3

{"height":1,"currentBlockHash":"RrndKwuojRMjOz/rdD7rJD/NUupiuBuCtQwnZG7Vdi/XXcTd2MDyAMsFAZ1ntZL2/IlcSUeatIZAKS6ss7fEvg=="}

[vagrant@ubuntu-1404 fabric]$

$ ./get_block.sh

vp0

{"height":2,"currentBlockHash":"YOZO7fZhVfUu+XPIyVxOWSZKePwJ/rVCm6uDtzP3RoGZQdtjzgJPZjgb+3vI7RldxhIg6PsxCEXw4N6tF18jeQ==","previousBlockHash":"RrndKwuojRMjOz/rdD7rJD/NUupiuBuCtQwnZG7Vdi/XXcTd2MDyAMsFAZ1ntZL2/IlcSUeatIZAKS6ss7fEvg=="}

vp1

{"height":2,"currentBlockHash":"YOZO7fZhVfUu+XPIyVxOWSZKePwJ/rVCm6uDtzP3RoGZQdtjzgJPZjgb+3vI7RldxhIg6PsxCEXw4N6tF18jeQ==","previousBlockHash":"RrndKwuojRMjOz/rdD7rJD/NUupiuBuCtQwnZG7Vdi/XXcTd2MDyAMsFAZ1ntZL2/IlcSUeatIZAKS6ss7fEvg=="}

vp2

{"height":2,"currentBlockHash":"YOZO7fZhVfUu+XPIyVxOWSZKePwJ/rVCm6uDtzP3RoGZQdtjzgJPZjgb+3vI7RldxhIg6PsxCEXw4N6tF18jeQ==","previousBlockHash":"RrndKwuojRMjOz/rdD7rJD/NUupiuBuCtQwnZG7Vdi/XXcTd2MDyAMsFAZ1ntZL2/IlcSUeatIZAKS6ss7fEvg=="}

vp3

{"height":2,"currentBlockHash":"YOZO7fZhVfUu+XPIyVxOWSZKePwJ/rVCm6uDtzP3RoGZQdtjzgJPZjgb+3vI7Rldxh
Ig6PsxCEXw4N6tF18jeQ==","previousBlockHash":"RrndKwuojRMjOz/rdD7rJD/NUupiuBuCtQwnZG7Vdi/XXcTd2MD
yAMsFAZ1ntZL2/IlcSUeatIZAKS6ss7fEvg=="}
[vagrant@ubuntu-1404 fabric]$

$ ./get_block.sh

vp0

{"height":3,"currentBlockHash":"A/7w4Rg2UsgL1req66R74+fj2+o6iu9p8Tg9lN5qZFATm9INMFoWPpzoruIJn2KY/K
fu0O/rH3oXadWR5Ww3cQ==","previousBlockHash":"YOZO7fZhVfUu+XPIyVxOWSZKePwJ/rVCm6uDtzP3RoGZQdt
jzgJPZjgb+3vI7RldxhIg6PsxCEXw4N6tF18jeQ=="}

vp1

{"height":3,"currentBlockHash":"A/7w4Rg2UsgL1req66R74+fj2+o6iu9p8Tg9lN5qZFATm9INMFoWPpzoruIJn2KY/K
fu0O/rH3oXadWR5Ww3cQ==","previousBlockHash":"YOZO7fZhVfUu+XPIyVxOWSZKePwJ/rVCm6uDtzP3RoGZQdt
jzgJPZjgb+3vI7RldxhIg6PsxCEXw4N6tF18jeQ=="}

vp2

{"height":3,"currentBlockHash":"A/7w4Rg2UsgL1req66R74+fj2+o6iu9p8Tg9lN5qZFATm9INMFoWPpzoruIJn2KY/K
fu0O/rH3oXadWR5Ww3cQ==","previousBlockHash":"YOZO7fZhVfUu+XPIyVxOWSZKePwJ/rVCm6uDtzP3RoGZQdt
jzgJPZjgb+3vI7RldxhIg6PsxCEXw4N6tF18jeQ=="}

vp3

{"height":3,"currentBlockHash":"A/7w4Rg2UsgL1req66R74+fj2+o6iu9p8Tg9lN5qZFATm9INMFoWPpzoruIJn2KY/K
fu0O/rH3oXadWR5Ww3cQ==","previousBlockHash":"YOZO7fZhVfUu+XPIyVxOWSZKePwJ/rVCm6uDtzP3RoGZQdt
jzgJPZjgb+3vI7RldxhIg6PsxCEXw4N6tF18jeQ=="}

[vagrant@ubuntu-1404 fabric]$

----------- vp1 stop -------------

$ ./get_block.sh

vp0

{"height":4,"currentBlockHash":"QEhM2tecsI2KWW8qHoWBiHoyoDIPWei7AU3k7Cn5tRHZxjlIGQ2gwg2+roHPhkec
X7dm50aKIjINnKJpYxgXXQ==","previousBlockHash":"A/7w4Rg2UsgL1req66R74+fj2+o6iu9p8Tg9lN5qZFATm9IN

MFoWPpzoruIJn2KY/Kfu0O/rH3oXadWR5Ww3cQ=="}

vp1

curl: (7) Failed to connect to 172.17.0.5 port 5000: No route to host

vp2

{"height":4,"currentBlockHash":"QEhM2tecsI2KWW8qHoWBiHoyoDIPWei7AU3k7Cn5tRHZxjlIGQ2gwg2+roHPhkec

X7dm50aKIjINnKJpYxgXXQ==","previousBlockHash":"A/7w4Rg2UsgL1req66R74+fj2+o6iu9p8Tg9lN5qZFATm9IN

MFoWPpzoruIJn2KY/Kfu0O/rH3oXadWR5Ww3cQ=="}

vp3

{"height":4,"currentBlockHash":"QEhM2tecsI2KWW8qHoWBiHoyoDIPWei7AU3k7Cn5tRHZxjlIGQ2gwg2+roHPhkec

X7dm50aKIjINnKJpYxgXXQ==","previousBlockHash":"A/7w4Rg2UsgL1req66R74+fj2+o6iu9p8Tg9lN5qZFATm9IN

MFoWPpzoruIJn2KY/Kfu0O/rH3oXadWR5Ww3cQ=="}

[vagrant@ubuntu-1404 fabric]$

----------- vp1 start -------------

$ ./get_block.sh

vp0

{"height":5,"currentBlockHash":"4h4q5vpy4hsbOyElJxhQBWM/e23UBrcAJR115LXLrXrW65VVDSmyfa8u+Vlrar+y

XifuFGAoJn3KOeOlEqYCuw==","previousBlockHash":"QEhM2tecsI2KWW8qHoWBiHoyoDIPWei7AU3k7Cn5tRHZxjlI

GQ2gwg2+roHPhkecX7dm50aKIjINnKJpYxgXXQ=="}

vp1

{"height":3,"currentBlockHash":"A/7w4Rg2UsgL1req66R74+fj2+o6iu9p8Tg9lN5qZFATm9INMFoWPpzoruIJn2KY/K

fu0O/rH3oXadWR5Ww3cQ==","previousBlockHash":"YOZO7fZhVfUu+XPIyVxOWSZKePwJ/rVCm6uDtzP3RoGZQdt

jzgJPZjgb+3vI7RldxhIg6PsxCEXw4N6tF18jeQ=="}

vp2

{"height":5,"currentBlockHash":"4h4q5vpy4hsbOyElJxhQBWM/e23UBrcAJR115LXLrXrW65VVDSmyfa8u+Vlrar+y

XifuFGAoJn3KOeOlEqYCuw==","previousBlockHash":"QEhM2tecsI2KWW8qHoWBiHoyoDIPWei7AU3k7Cn5tRHZxjlI

GQ2gwg2+roHPhkecX7dm50aKIjINnKJpYxgXXQ=="}

vp3

{"height":5,"currentBlockHash":"4h4q5vpy4hsbOyElJxhQBWM/e23UBrcAJR115LXLrXrW65VVDSmyfa8u+Vlrar+y

XifuFGAoJn3KOeOlEqYCuw==","previousBlockHash":"QEhM2tecsI2KWW8qHoWBiHoyoDIPWei7AU3k7Cn5tRHZxjlI

GQ2gwg2+roHPhkecX7dm50aKIjINnKJpYxgXXQ=="}

[vagrant@ubuntu-1404 fabric]$

$ ./get_block.sh

vp0

{"height":6,"currentBlockHash":"hu3mkGjf/RDQrZ5ta8FFfnx0dWoFGARek4/2TNBAPv891utbbQktOL4TKgpTGMhA

Lf7ExdI2nFFa5Vj5Fvh8Iw==","previousBlockHash":"4h4q5vpy4hsbOyElJxhQBWM/e23UBrcAJR115LXLrXrW65VVD

Smyfa8u+Vlrar+yXifuFGAoJn3KOeOlEqYCuw=="}

vp1

{"height":3,"currentBlockHash":"A/7w4Rg2UsgL1req66R74+fj2+o6iu9p8Tg9lN5qZFATm9INMFoWPpzoruIJn2KY/K

fu0O/rH3oXadWR5Ww3cQ==","previousBlockHash":"YOZO7fZhVfUu+XPIyVxOWSZKePwJ/rVCm6uDtzP3RoGZQdt

jzgJPZjgb+3vI7RldxhIg6PsxCEXw4N6tF18jeQ=="}

vp2

{"height":6,"currentBlockHash":"hu3mkGjf/RDQrZ5ta8FFfnx0dWoFGARek4/2TNBAPv891utbbQktOL4TKgpTGMhA

Lf7ExdI2nFFa5Vj5Fvh8Iw==","previousBlockHash":"4h4q5vpy4hsbOyElJxhQBWM/e23UBrcAJR115LXLrXrW65VVD

Smyfa8u+Vlrar+yXifuFGAoJn3KOeOlEqYCuw=="}

vp3

{"height":6,"currentBlockHash":"hu3mkGjf/RDQrZ5ta8FFfnx0dWoFGARek4/2TNBAPv891utbbQktOL4TKgpTGMhA

Lf7ExdI2nFFa5Vj5Fvh8Iw==","previousBlockHash":"4h4q5vpy4hsbOyElJxhQBWM/e23UBrcAJR115LXLrXrW65VVD

Smyfa8u+Vlrar+yXifuFGAoJn3KOeOlEqYCuw=="}

[vagrant@ubuntu-1404 fabric]$

$ ./get_block.sh

vp0

{"height":7,"currentBlockHash":"R0896ePWRW6fBbf0IX0nx8c4wc6zhIJE27JLEuk5H7bUg3VBpn/gcgAu8nqFY6Wye

lkzaamqwZiophJtmGeBeA==","previousBlockHash":"hu3mkGjf/RDQrZ5ta8FFfnx0dWoFGARek4/2TNBAPv891utbb

QktOL4TKgpTGMhALf7ExdI2nFFa5Vj5Fvh8Iw=="}

vp1

{"height":3,"currentBlockHash":"A/7w4Rg2UsgL1req66R74+fj2+o6iu9p8Tg9lN5qZFATm9INMFoWPpzoruIJn2KY/K

fu0O/rH3oXadWR5Ww3cQ==","previousBlockHash":"YOZO7fZhVfUu+XPIyVxOWSZKePwJ/rVCm6uDtzP3RoGZQdt

jzgJPZjgb+3vI7RldxhIg6PsxCEXw4N6tF18jeQ=="}

vp2

{"height":7,"currentBlockHash":"R0896ePWRW6fBbf0IX0nx8c4wc6zhIJE27JLEuk5H7bUg3VBpn/gcgAu8nqFY6Wye

lkzaamqwZiophJtmGeBeA==","previousBlockHash":"hu3mkGjf/RDQrZ5ta8FFfnx0dWoFGARek4/2TNBAPv891utbb

QktOL4TKgpTGMhALf7ExdI2nFFa5Vj5Fvh8Iw=="}

vp3

{"height":7,"currentBlockHash":"R0896ePWRW6fBbf0IX0nx8c4wc6zhIJE27JLEuk5H7bUg3VBpn/gcgAu8nqFY6Wye

lkzaamqwZiophJtmGeBeA==","previousBlockHash":"hu3mkGjf/RDQrZ5ta8FFfnx0dWoFGARek4/2TNBAPv891utbb

QktOL4TKgpTGMhALf7ExdI2nFFa5Vj5Fvh8Iw=="}

[vagrant@ubuntu-1404 fabric]$

$ ./get_block.sh

vp0

{"height":8,"currentBlockHash":"CYdhLTgDvJz7gBB8ZaCenXEdRwRPBhhlF+vclLNEfgi04ygchKDbb3VBE7jqCmz0v

OOHHsOYZvgbLrQPyQ+bvg==","previousBlockHash":"R0896ePWRW6fBbf0IX0nx8c4wc6zhIJE27JLEuk5H7bUg3V

Bpn/gcgAu8nqFY6WyelkzaamqwZiophJtmGeBeA=="}

vp1

{"height":3,"currentBlockHash":"A/7w4Rg2UsgL1req66R74+fj2+o6iu9p8Tg9lN5qZFATm9INMFoWPpzoruIJn2KY/K

fu0O/rH3oXadWR5Ww3cQ==","previousBlockHash":"YOZO7fZhVfUu+XPIyVxOWSZKePwJ/rVCm6uDtzP3RoGZQdt

jzgJPZjgb+3vI7RldxhIg6PsxCEXw4N6tF18jeQ=="}

vp2

{"height":8,"currentBlockHash":"CYdhLTgDvJz7gBB8ZaCenXEdRwRPBhhlF+vclLNEfgi04ygchKDbb3VBE7jqCmz0v

OOHHsOYZvgbLrQPyQ+bvg==","previousBlockHash":"R0896ePWRW6fBbf0IX0nx8c4wc6zhIJE27JLEuk5H7bUg3V

Bpn/gcgAu8nqFY6WyelkzaamqwZiophJtmGeBeA=="}

vp3

{"height":8,"currentBlockHash":"CYdhLTgDvJz7gBB8ZaCenXEdRwRPBhhlF+vclLNEfgi04ygchKDbb3VBE7jqCmz0v

OOHHsOYZvgbLrQPyQ+bvg==","previousBlockHash":"R0896ePWRW6fBbf0IX0nx8c4wc6zhIJE27JLEuk5H7bUg3V

Bpn/gcgAu8nqFY6WyelkzaamqwZiophJtmGeBeA=="}

[vagrant@ubuntu-1404 fabric]$

$ ./get_block.sh

vp0

{"height":9,"currentBlockHash":"X3LxiedC66I9bRFgekBL2pikCNKjIueGeSddOTwxcibDAGTNwIarR3k33fLYYLU4Gy
G0QmESyBXHQl0gv7CJ+A==","previousBlockHash":"CYdhLTgDvJz7gBB8ZaCenXEdRwRPBhhlF+vclLNEfgi04ygch
KDbb3VBE7jqCmz0vOOHHsOYZvgbLrQPyQ+bvg=="}

vp1

{"height":9,"currentBlockHash":"X3LxiedC66I9bRFgekBL2pikCNKjIueGeSddOTwxcibDAGTNwIarR3k33fLYYLU4Gy
G0QmESyBXHQl0gv7CJ+A==","previousBlockHash":"CYdhLTgDvJz7gBB8ZaCenXEdRwRPBhhlF+vclLNEfgi04ygch
KDbb3VBE7jqCmz0vOOHHsOYZvgbLrQPyQ+bvg=="}

vp2

{"height":9,"currentBlockHash":"X3LxiedC66I9bRFgekBL2pikCNKjIueGeSddOTwxcibDAGTNwIarR3k33fLYYLU4Gy
G0QmESyBXHQl0gv7CJ+A==","previousBlockHash":"CYdhLTgDvJz7gBB8ZaCenXEdRwRPBhhlF+vclLNEfgi04ygch
KDbb3VBE7jqCmz0vOOHHsOYZvgbLrQPyQ+bvg=="}

vp3

{"height":9,"currentBlockHash":"X3LxiedC66I9bRFgekBL2pikCNKjIueGeSddOTwxcibDAGTNwIarR3k33fLYYLU4Gy
G0QmESyBXHQl0gv7CJ+A==","previousBlockHash":"CYdhLTgDvJz7gBB8ZaCenXEdRwRPBhhlF+vclLNEfgi04ygch
KDbb3VBE7jqCmz0vOOHHsOYZvgbLrQPyQ+bvg=="}

[vagrant@ubuntu-1404 fabric]$

$ ./get_block.sh

vp0

{"height":10,"currentBlockHash":"DXJ8xMW6HAZ2tMfkUgyNZAqGo/Cuufy4axnQPBID3Kk+phTmb4zZsrDvCcMkQi
ElbYFrn2Pyoc5las/D9PC0bQ==","previousBlockHash":"X3LxiedC66I9bRFgekBL2pikCNKjIueGeSddOTwxcibDAGTN
wIarR3k33fLYYLU4GyG0QmESyBXHQl0gv7CJ+A=="}

vp1

{"height":9,"currentBlockHash":"X3LxiedC66I9bRFgekBL2pikCNKjIueGeSddOTwxcibDAGTNwIarR3k33fLYYLU4Gy
G0QmESyBXHQl0gv7CJ+A==","previousBlockHash":"CYdhLTgDvJz7gBB8ZaCenXEdRwRPBhhlF+vclLNEfgi04ygch
KDbb3VBE7jqCmz0vOOHHsOYZvgbLrQPyQ+bvg=="}

vp2

{"height":10,"currentBlockHash":"DXJ8xMW6HAZ2tMfkUgyNZAqGo/Cuufy4axnQPBID3Kk+phTmb4zZsrDvCcMkQi
ElbYFrn2Pyoc5las/D9PC0bQ==","previousBlockHash":"X3LxiedC66I9bRFgekBL2pikCNKjIueGeSddOTwxcibDAGTN
wIarR3k33fLYYLU4GyG0QmESyBXHQl0gv7CJ+A=="}

vp3

{"height":10,"currentBlockHash":"DXJ8xMW6HAZ2tMfkUgyNZAqGo/Cuufy4axnQPBID3Kk+phTmb4zZsrDvCcMkQi

ElbYFrn2Pyoc5las/D9PC0bQ==","previousBlockHash":"X3LxiedC66I9bRFgekBL2pikCNKjIueGeSddOTwxcibDAGTN

wlarR3k33fLYYLU4GyG0QmESyBXHQl0gv7CJ+A=="}

[vagrant@ubuntu-1404 fabric]$

]$ ./get_block.sh

vp0

{"height":11,"currentBlockHash":"VA3JVfvwKWCP7+jUfDvcAG5p9l/bRdTqPPwcPPwf/wtTOg+yIwM+2AwRCRPQAw

0MBBRvazBDbGiiABSsAsu28A==","previousBlockHash":"DXJ8xMW6HAZ2tMfkUgyNZAqGo/Cuufy4axnQPBID3Kk

+phTmb4zZsrDvCcMkQiElbYFrn2Pyoc5las/D9PC0bQ=="}

vp1

{"height":9,"currentBlockHash":"X3LxiedC66I9bRFgekBL2pikCNKjIueGeSddOTwxcibDAGTNwlarR3k33fLYYLU4Gy

G0QmESyBXHQl0gv7CJ+A==","previousBlockHash":"CYdhLTgDvJz7gBB8ZaCenXEdRwRPBhhlF+vclLNEfgi04ygch

KDbb3VBE7jqCmz0vOOHHsOYZvgbLrQPyQ+bvg=="}

vp2

{"height":11,"currentBlockHash":"VA3JVfvwKWCP7+jUfDvcAG5p9l/bRdTqPPwcPPwf/wtTOg+yIwM+2AwRCRPQAw

0MBBRvazBDbGiiABSsAsu28A==","previousBlockHash":"DXJ8xMW6HAZ2tMfkUgyNZAqGo/Cuufy4axnQPBID3Kk

+phTmb4zZsrDvCcMkQiElbYFrn2Pyoc5las/D9PC0bQ=="}

vp3

{"height":11,"currentBlockHash":"VA3JVfvwKWCP7+jUfDvcAG5p9l/bRdTqPPwcPPwf/wtTOg+yIwM+2AwRCRPQAw

0MBBRvazBDbGiiABSsAsu28A==","previousBlockHash":"DXJ8xMW6HAZ2tMfkUgyNZAqGo/Cuufy4axnQPBID3Kk

+phTmb4zZsrDvCcMkQiElbYFrn2Pyoc5las/D9PC0bQ=="}

[vagrant@ubuntu-1404 fabric]$

$ ./get_block.sh

vp0

{"height":12,"currentBlockHash":"ggMOHYpbcz4aQCE9xXRwxSlpcdu/Xv29/EDbSmz2iiX5j5jDqnJ0Und4GP+U+KZ

eQscO/LvkkM3sp1MhtjQvmQ==","previousBlockHash":"VA3JVfvwKWCP7+jUfDvcAG5p9l/bRdTqPPwcPPwf/wtTOg

+yIwM+2AwRCRPQAw0MBBRvazBDbGiiABSsAsu28A=="}

vp1

{"height":9,"currentBlockHash":"X3LxiedC66I9bRFgekBL2pikCNKjIueGeSddOTwxcibDAGTNwlarR3k33fLYYLU4Gy
G0QmESyBXHQl0gv7CJ+A==","previousBlockHash":"CYdhLTgDvJz7gBB8ZaCenXEdRwRPBhhlF+vclLNEfgi04ygch
KDbb3VBE7jqCmz0vOOHHsOYZvgbLrQPyQ+bvg=="}

vp2

{"height":12,"currentBlockHash":"ggMOHYpbcz4aQCE9xXRwxSlpcdu/Xv29/EDbSmz2iiX5j5jDqnJ0Und4GP+U+KZ
eQscO/LvkkM3sp1MhtjQvmQ==","previousBlockHash":"VA3JVfvwKWCP7+jUfDvcAG5p9l/bRdTqPPwcPPwf/wtTOg
+yIwM+2AwRCRPQAw0MBBRvazBDbGiiABSsAsu28A=="}

vp3

{"height":12,"currentBlockHash":"ggMOHYpbcz4aQCE9xXRwxSlpcdu/Xv29/EDbSmz2iiX5j5jDqnJ0Und4GP+U+KZ
eQscO/LvkkM3sp1MhtjQvmQ==","previousBlockHash":"VA3JVfvwKWCP7+jUfDvcAG5p9l/bRdTqPPwcPPwf/wtTOg
+yIwM+2AwRCRPQAw0MBBRvazBDbGiiABSsAsu28A=="}

[vagrant@ubuntu-1404 fabric]$

$ ./get_block.sh

vp0

{"height":13,"currentBlockHash":"QYZQy0ZC75y1EBAUdnigik3ywaxY4j4o/IM+dDhRcCSAarYkellA9XTZ7dpL4o6m
lCcjpZT5uPCXIX18J+bfTA==","previousBlockHash":"ggMOHYpbcz4aQCE9xXRwxSlpcdu/Xv29/EDbSmz2iiX5j5jDq
nJ0Und4GP+U+KZeQscO/LvkkM3sp1MhtjQvmQ=="}

vp1

{"height":9,"currentBlockHash":"X3LxiedC66I9bRFgekBL2pikCNKjIueGeSddOTwxcibDAGTNwlarR3k33fLYYLU4Gy
G0QmESyBXHQl0gv7CJ+A==","previousBlockHash":"CYdhLTgDvJz7gBB8ZaCenXEdRwRPBhhlF+vclLNEfgi04ygch
KDbb3VBE7jqCmz0vOOHHsOYZvgbLrQPyQ+bvg=="}

vp2

{"height":13,"currentBlockHash":"QYZQy0ZC75y1EBAUdnigik3ywaxY4j4o/IM+dDhRcCSAarYkellA9XTZ7dpL4o6m
lCcjpZT5uPCXIX18J+bfTA==","previousBlockHash":"ggMOHYpbcz4aQCE9xXRwxSlpcdu/Xv29/EDbSmz2iiX5j5jDq
nJ0Und4GP+U+KZeQscO/LvkkM3sp1MhtjQvmQ=="}

vp3

{"height":13,"currentBlockHash":"QYZQy0ZC75y1EBAUdnigik3ywaxY4j4o/IM+dDhRcCSAarYkellA9XTZ7dpL4o6m
lCcjpZT5uPCXIX18J+bfTA==","previousBlockHash":"ggMOHYpbcz4aQCE9xXRwxSlpcdu/Xv29/EDbSmz2iiX5j5jDq
nJ0Und4GP+U+KZeQscO/LvkkM3sp1MhtjQvmQ=="}

[vagrant@ubuntu-1404 fabric]$

$ ./get_block.sh

vp0

{"height":14,"currentBlockHash":"jQak3rVCzJ8Bo/oeiieUCv6EpRs+prvYiWxNBZEwSfGPPmqNzBxOqaI7+EFHz1/ky

PVIQTmUTmafs2L30ZdGug==","previousBlockHash":"QYZQy0ZC75y1EBAUdnigik3ywaxY4j4o/IM+dDhRcCSAarY

kellA9XTZ7dpL4o6mlCcjpZT5uPCXIX18J+bfTA=="}

vp1

{"height":9,"currentBlockHash":"X3LxiedC66I9bRFgekBL2pikCNKjIueGeSddOTwxcibDAGTNwlarR3k33fLYYLU4Gy

G0QmESyBXHQl0gv7CJ+A==","previousBlockHash":"CYdhLTgDvJz7gBB8ZaCenXEdRwRPBhhlF+vcILNEfgi04ygch

KDbb3VBE7jqCmz0vOOHHsOYZvgbLrQPyQ+bvg=="}

vp2

{"height":14,"currentBlockHash":"jQak3rVCzJ8Bo/oeiieUCv6EpRs+prvYiWxNBZEwSfGPPmqNzBxOqaI7+EFHz1/ky

PVIQTmUTmafs2L30ZdGug==","previousBlockHash":"QYZQy0ZC75y1EBAUdnigik3ywaxY4j4o/IM+dDhRcCSAarY

kellA9XTZ7dpL4o6mlCcjpZT5uPCXIX18J+bfTA=="}

vp3

{"height":14,"currentBlockHash":"jQak3rVCzJ8Bo/oeiieUCv6EpRs+prvYiWxNBZEwSfGPPmqNzBxOqaI7+EFHz1/ky

PVIQTmUTmafs2L30ZdGug==","previousBlockHash":"QYZQy0ZC75y1EBAUdnigik3ywaxY4j4o/IM+dDhRcCSAarY

kellA9XTZ7dpL4o6mlCcjpZT5uPCXIX18J+bfTA=="}

[vagrant@ubuntu-1404 fabric]$

[vagrant@ubuntu-1404 fabric]$

$ ./get_block.sh

vp0

{"height":15,"currentBlockHash":"A24fymYzY2fQwwuY/z73dY/pCi90OpaUIDiWz4M4Z9qoiAAAK/bxle5qQY7lKFI1g

3HDKuvQjc+3rvAsilivVA==","previousBlockHash":"jQak3rVCzJ8Bo/oeiieUCv6EpRs+prvYiWxNBZEwSfGPPmqNzBx

OqaI7+EFHz1/kyPVIQTmUTmafs2L30ZdGug=="}

vp1

{"height":15,"currentBlockHash":"A24fymYzY2fQwwuY/z73dY/pCi90OpaUIDiWz4M4Z9qoiAAAK/bxle5qQY7lKFI1g

3HDKuvQjc+3rvAsilivVA==","previousBlockHash":"jQak3rVCzJ8Bo/oeiieUCv6EpRs+prvYiWxNBZEwSfGPPmqNzBx

OqaI7+EFHz1/kyPVIQTmUTmafs2L30ZdGug=="}

vp2

{"height":15,"currentBlockHash":"A24fymYzY2fQwwuY/z73dY/pCi90OpaUIDiWz4M4Z9qoiAAAK/bxle5qQY7lKFI1g
3HDKuvQjc+3rvAsilivVA==","previousBlockHash":"jQak3rVCzJ8Bo/oeiieUCv6EpRs+prvYiWxNBZEwSfGPPmqNzBx
OqaI7+EFHz1/kyPVIQTmUTmafs2L30ZdGug=="}

vp3

{"height":15,"currentBlockHash":"A24fymYzY2fQwwuY/z73dY/pCi90OpaUIDiWz4M4Z9qoiAAAK/bxle5qQY7lKFI1g
3HDKuvQjc+3rvAsilivVA==","previousBlockHash":"jQak3rVCzJ8Bo/oeiieUCv6EpRs+prvYiWxNBZEwSfGPPmqNzBx
OqaI7+EFHz1/kyPVIQTmUTmafs2L30ZdGug=="}

[vagrant@ubuntu-1404 fabric]$

$ ./get_block.sh

vp0

{"height":16,"currentBlockHash":"jBU+ct82OfnQCAY1sOav8kZ3B40X2ZDvBks1J37pDoERU1yxPt7NASCB493jr43/
/kUF324Z+n6KZ1uYEBug/w==","previousBlockHash":"A24fymYzY2fQwwuY/z73dY/pCi90OpaUIDiWz4M4Z9qoiAA
AK/bxle5qQY7lKFI1g3HDKuvQjc+3rvAsilivVA=="}

vp1

{"height":15,"currentBlockHash":"A24fymYzY2fQwwuY/z73dY/pCi90OpaUIDiWz4M4Z9qoiAAAK/bxle5qQY7lKFI1g
3HDKuvQjc+3rvAsilivVA==","previousBlockHash":"jQak3rVCzJ8Bo/oeiieUCv6EpRs+prvYiWxNBZEwSfGPPmqNzBx
OqaI7+EFHz1/kyPVIQTmUTmafs2L30ZdGug=="}

vp2

{"height":16,"currentBlockHash":"jBU+ct82OfnQCAY1sOav8kZ3B40X2ZDvBks1J37pDoERU1yxPt7NASCB493jr43/
/kUF324Z+n6KZ1uYEBug/w==","previousBlockHash":"A24fymYzY2fQwwuY/z73dY/pCi90OpaUIDiWz4M4Z9qoiAA
AK/bxle5qQY7lKFI1g3HDKuvQjc+3rvAsilivVA=="}

vp3

{"height":16,"currentBlockHash":"jBU+ct82OfnQCAY1sOav8kZ3B40X2ZDvBks1J37pDoERU1yxPt7NASCB493jr43/
/kUF324Z+n6KZ1uYEBug/w==","previousBlockHash":"A24fymYzY2fQwwuY/z73dY/pCi90OpaUIDiWz4M4Z9qoiAA
AK/bxle5qQY7lKFI1g3HDKuvQjc+3rvAsilivVA=="}

[vagrant@ubuntu-1404 fabric]$

----------vp2 stop -----------

$ ./get_block.

sh

vp0

{"height":16,"currentBlockHash":"jBU+ct82OfnQCAY1sOav8kZ3B40X2ZDvBks1J37pDoERU1yxPt7NASCB493jr43/

/kUF324Z+n6KZ1uYEBug/w==","previousBlockHash":"A24fymYzY2fQwwuY/z73dY/pCi90OpaUIDiWz4M4Z9qoiAA

AK/bxle5qQY7lKFI1g3HDKuvQjc+3rvAsilivVA=="}

vp1

{"height":15,"currentBlockHash":"A24fymYzY2fQwwuY/z73dY/pCi90OpaUIDiWz4M4Z9qoiAAAK/bxle5qQY7lKFI1g

3HDKuvQjc+3rvAsilivVA==","previousBlockHash":"jQak3rVCzJ8Bo/oeiieUCv6EpRs+prvYiWxNBZEwSfGPPmqNzBx

OqaI7+EFHz1/kyPVIQTmUTmafs2L30ZdGug=="}

vp2

curl: (7) Failed to connect to 172.17.0.6 port 5000: No route to host

vp3

{"height":16,"currentBlockHash":"jBU+ct82OfnQCAY1sOav8kZ3B40X2ZDvBks1J37pDoERU1yxPt7NASCB493jr43/

/kUF324Z+n6KZ1uYEBug/w==","previousBlockHash":"A24fymYzY2fQwwuY/z73dY/pCi90OpaUIDiWz4M4Z9qoiAA

AK/bxle5qQY7lKFI1g3HDKuvQjc+3rvAsilivVA=="}

[vagrant@ubuntu-1404 fabric]$

----------vp2 start -----------

====before invoke

$ ./get_block.sh

vp0

{"height":17,"currentBlockHash":"hWbu6QQEXU2WcD8ZS3wlZiAAFXHLh788Tt7+HbVuplkbRsOL9y2VT0t0EyOB

mEKone6rIMUXqXK4eCqoXN9+fg==","previousBlockHash":"jBU+ct82OfnQCAY1sOav8kZ3B40X2ZDvBks1J37pDo

ERU1yxPt7NASCB493jr43//kUF324Z+n6KZ1uYEBug/w=="}

vp1

{"height":17,"currentBlockHash":"hWbu6QQEXU2WcD8ZS3wlZiAAFXHLh788Tt7+HbVuplkbRsOL9y2VT0t0EyOB

mEKone6rIMUXqXK4eCqoXN9+fg==","previousBlockHash":"jBU+ct82OfnQCAY1sOav8kZ3B40X2ZDvBks1J37pDo

ERU1yxPt7NASCB493jr43//kUF324Z+n6KZ1uYEBug/w=="}

vp2

{"height":16,"currentBlockHash":"jBU+ct82OfnQCAY1sOav8kZ3B40X2ZDvBks1J37pDoERU1yxPt7NASCB493jr43/

/kUF324Z+n6KZ1uYEBug/w==","previousBlockHash":"A24fymYzY2fQwwuY/z73dY/pCi90OpaUIDiWz4M4Z9qoiAA

AK/bxle5qQY7lKFI1g3HDKuvQjc+3rvAsilivVA=="}

vp3

{"height":17,"currentBlockHash":"hWbu6QQEXU2WcD8ZS3wlZiAAFXHLh788Tt7+HbVuplkbRsOL9y2VT0t0EyOB

mEKone6rIMUXqXK4eCqoXN9+fg==","previousBlockHash":"jBU+ct82OfnQCAY1sOav8kZ3B40X2ZDvBks1J37pDo

ERU1yxPt7NASCB493jr43//kUF324Z+n6KZ1uYEBug/w=="}

[vagrant@ubuntu-1404 fabric]$

=====after invoke

$ ./get_block.sh

vp0

{"height":18,"currentBlockHash":"z3WWJHYte5Y7es1PNj4ro6ixo2WVd+5ZhJDWAg/uinECR3EYiH1saRgYoeeUz0yq

glaiGAd0UdQCFRAB80uJtg==","previousBlockHash":"hWbu6QQEXU2WcD8ZS3wlZiAAFXHLh788Tt7+HbVuplkbRs

OL9y2VT0t0EyOBmEKone6rIMUXqXK4eCqoXN9+fg=="}

vp1

{"height":18,"currentBlockHash":"z3WWJHYte5Y7es1PNj4ro6ixo2WVd+5ZhJDWAg/uinECR3EYiH1saRgYoeeUz0yq

glaiGAd0UdQCFRAB80uJtg==","previousBlockHash":"hWbu6QQEXU2WcD8ZS3wlZiAAFXHLh788Tt7+HbVuplkbRs

OL9y2VT0t0EyOBmEKone6rIMUXqXK4eCqoXN9+fg=="}

vp2

{"height":16,"currentBlockHash":"jBU+ct82OfnQCAY1sOav8kZ3B40X2ZDvBks1J37pDoERU1yxPt7NASCB493jr43/

/kUF324Z+n6KZ1uYEBug/w==","previousBlockHash":"A24fymYzY2fQwwuY/z73dY/pCi90OpaUIDiWz4M4Z9qoiAA

AK/bxle5qQY7lKFI1g3HDKuvQjc+3rvAsilivVA=="}

vp3

{"height":18,"currentBlockHash":"z3WWJHYte5Y7es1PNj4ro6ixo2WVd+5ZhJDWAg/uinECR3EYiH1saRgYoeeUz0yq

glaiGAd0UdQCFRAB80uJtg==","previousBlockHash":"hWbu6QQEXU2WcD8ZS3wlZiAAFXHLh788Tt7+HbVuplkbRs

OL9y2VT0t0EyOBmEKone6rIMUXqXK4eCqoXN9+fg=="}

[vagrant@ubuntu-1404 fabric]$

```
$ ./get_block.sh

vp0

{"height":19,"currentBlockHash":"mTJtFgrWkkxRvvoBKNrVG7Mz1C3Kt4VRJHQ4SZLsN/3tcQMWM8L7lMsDuXzepV
a+r6jSk8JZdkmC1PbMYGoqnw==","previousBlockHash":"z3WWJHYte5Y7es1PNj4ro6ixo2WVd+5ZhJDWAg/uinEC
R3EYiH1saRgYoeeUz0yqgIaiGAd0UdQCFRAB80uJtg=="}

vp1

{"height":19,"currentBlockHash":"mTJtFgrWkkxRvvoBKNrVG7Mz1C3Kt4VRJHQ4SZLsN/3tcQMWM8L7lMsDuXzepV
a+r6jSk8JZdkmC1PbMYGoqnw==","previousBlockHash":"z3WWJHYte5Y7es1PNj4ro6ixo2WVd+5ZhJDWAg/uinEC
R3EYiH1saRgYoeeUz0yqgIaiGAd0UdQCFRAB80uJtg=="}

vp2

{"height":16,"currentBlockHash":"jBU+ct82OfnQCAY1sOav8kZ3B40X2ZDvBks1J37pDoERU1yxPt7NASCB493jr43/
/kUF324Z+n6KZ1uYEBug/w==","previousBlockHash":"A24fymYzY2fQwwuY/z73dY/pCi90OpaUIDiWz4M4Z9qoiAA
AK/bxle5qQY7lKFI1g3HDKuvQjc+3rvAsilivVA=="}

vp3

{"height":19,"currentBlockHash":"mTJtFgrWkkxRvvoBKNrVG7Mz1C3Kt4VRJHQ4SZLsN/3tcQMWM8L7lMsDuXzepV
a+r6jSk8JZdkmC1PbMYGoqnw==","previousBlockHash":"z3WWJHYte5Y7es1PNj4ro6ixo2WVd+5ZhJDWAg/uinEC
R3EYiH1saRgYoeeUz0yqgIaiGAd0UdQCFRAB80uJtg=="}

[vagrant@ubuntu-1404 fabric]$
```

```
$ ./get_block.sh

vp0

{"height":20,"currentBlockHash":"Rdat/CfO/11ULfcjpX+tOTlB76+6YsZwmijdfk7CT8qwF9DMNye25Cz0xAUpDrue
KYM1EyLdS9H+zNR4fWXf/g==","previousBlockHash":"mTJtFgrWkkxRvvoBKNrVG7Mz1C3Kt4VRJHQ4SZLsN/3tcQ
MWM8L7lMsDuXzepVa+r6jSk8JZdkmC1PbMYGoqnw=="}

vp1

{"height":20,"currentBlockHash":"Rdat/CfO/11ULfcjpX+tOTlB76+6YsZwmijdfk7CT8qwF9DMNye25Cz0xAUpDrue
KYM1EyLdS9H+zNR4fWXf/g==","previousBlockHash":"mTJtFgrWkkxRvvoBKNrVG7Mz1C3Kt4VRJHQ4SZLsN/3tcQ
MWM8L7lMsDuXzepVa+r6jSk8JZdkmC1PbMYGoqnw=="}

vp2
```

{"height":20,"currentBlockHash":"Rdat/CfO/11ULfcjpX+tOTlB76+6YsZwmijdfk7CT8qwF9DMNye25Cz0xAUpDrue
KYM1EyLdS9H+zNR4fWXf/g==","previousBlockHash":"mTJtFgrWkkxRvvoBKNrVG7Mz1C3Kt4VRJHQ4SZLsN/3tcQ
MWM8L7lMsDuXzepVa+r6jSk8JZdkmC1PbMYGoqnw=="}

vp3

{"height":20,"currentBlockHash":"Rdat/CfO/11ULfcjpX+tOTlB76+6YsZwmijdfk7CT8qwF9DMNye25Cz0xAUpDrue
KYM1EyLdS9H+zNR4fWXf/g==","previousBlockHash":"mTJtFgrWkkxRvvoBKNrVG7Mz1C3Kt4VRJHQ4SZLsN/3tcQ
MWM8L7lMsDuXzepVa+r6jSk8JZdkmC1PbMYGoqnw=="}

[vagrant@ubuntu-1404 fabric]$

$ ./get_block.sh

vp0

{"height":21,"currentBlockHash":"eJiaUWYteAiQ2O+XI+KagcbAX4/Mjtb1Kr/eReIpZQsMF2nsv6N+qBdR+hkqlYRlS
6ldyTRX/WifQFsxHozeTg==","previousBlockHash":"Rdat/CfO/11ULfcjpX+tOTlB76+6YsZwmijdfk7CT8qwF9DMNy
e25Cz0xAUpDrueKYM1EyLdS9H+zNR4fWXf/g=="}

vp1

{"height":21,"currentBlockHash":"eJiaUWYteAiQ2O+XI+KagcbAX4/Mjtb1Kr/eReIpZQsMF2nsv6N+qBdR+hkqlYRlS
6ldyTRX/WifQFsxHozeTg==","previousBlockHash":"Rdat/CfO/11ULfcjpX+tOTlB76+6YsZwmijdfk7CT8qwF9DMNy
e25Cz0xAUpDrueKYM1EyLdS9H+zNR4fWXf/g=="}

vp2

{"height":20,"currentBlockHash":"Rdat/CfO/11ULfcjpX+tOTlB76+6YsZwmijdfk7CT8qwF9DMNye25Cz0xAUpDrue
KYM1EyLdS9H+zNR4fWXf/g==","previousBlockHash":"mTJtFgrWkkxRvvoBKNrVG7Mz1C3Kt4VRJHQ4SZLsN/3tcQ
MWM8L7lMsDuXzepVa+r6jSk8JZdkmC1PbMYGoqnw=="}

vp3

{"height":21,"currentBlockHash":"eJiaUWYteAiQ2O+XI+KagcbAX4/Mjtb1Kr/eReIpZQsMF2nsv6N+qBdR+hkqlYRlS
6ldyTRX/WifQFsxHozeTg==","previousBlockHash":"Rdat/CfO/11ULfcjpX+tOTlB76+6YsZwmijdfk7CT8qwF9DMNy
e25Cz0xAUpDrueKYM1EyLdS9H+zNR4fWXf/g=="}

[vagrant@ubuntu-1404 fabric]$

$ ./get_block.sh

vp0

{"height":22,"currentBlockHash":"g8ccLkI0dJH27kZ3O5Ldh9/6c84KNZU0jetpzN1tLlxG9aWTyECZM9NQ02ogar5Z

k3si/Zdl7rYI+EFhufhXKw==","previousBlockHash":"eJiaUWYteAiQ2O+XI+KagcbAX4/Mjtb1Kr/eReIpZQsMF2nsv6N

+qBdR+hkqlYRlS6ldyTRX/WifQFsxHozeTg=="}

vp1

{"height":22,"currentBlockHash":"g8ccLkI0dJH27kZ3O5Ldh9/6c84KNZU0jetpzN1tLlxG9aWTyECZM9NQ02ogar5Z

k3si/Zdl7rYI+EFhufhXKw==","previousBlockHash":"eJiaUWYteAiQ2O+XI+KagcbAX4/Mjtb1Kr/eReIpZQsMF2nsv6N

+qBdR+hkqlYRlS6ldyTRX/WifQFsxHozeTg=="}

vp2

{"height":20,"currentBlockHash":"Rdat/CfO/11ULfcjpX+tOTlB76+6YsZwmijdfk7CT8qwF9DMNye25Cz0xAUpDrue

KYM1EyLdS9H+zNR4fWXf/g==","previousBlockHash":"mTJtFgrWkkxRvvoBKNrVG7Mz1C3Kt4VRJHQ4SZLsN/3tcQ

MWM8L7lMsDuXzepVa+r6jSk8JZdkmC1PbMYGoqnw=="}

vp3

{"height":22,"currentBlockHash":"g8ccLkI0dJH27kZ3O5Ldh9/6c84KNZU0jetpzN1tLlxG9aWTyECZM9NQ02ogar5Z

k3si/Zdl7rYI+EFhufhXKw==","previousBlockHash":"eJiaUWYteAiQ2O+XI+KagcbAX4/Mjtb1Kr/eReIpZQsMF2nsv6N

+qBdR+hkqlYRlS6ldyTRX/WifQFsxHozeTg=="}

[vagrant@ubuntu-1404 fabric]$

[vagrant@ubuntu-1404 fabric]$

$ ./get_block.sh

vp0

{"height":24,"currentBlockHash":"Dcy3znnXDx3C79j+JYtY0K5peVSIXWs/3rCiCo49hmXOboqZPXFrLCKCCAzW6t8

3zwmFFGxyJDL3TUYh8z3Kxg==","previousBlockHash":"G2TYiTDZ+vu9Pr1ZEtlBMyEW8T4aGyM4sw/ZGrfLwtA/V7

uRh/+/mWeoyvNQxx80dQJ3Jy4UIbnHFOBND/4Skg=="}

vp1

{"height":24,"currentBlockHash":"Dcy3znnXDx3C79j+JYtY0K5peVSIXWs/3rCiCo49hmXOboqZPXFrLCKCCAzW6t8

3zwmFFGxyJDL3TUYh8z3Kxg==","previousBlockHash":"G2TYiTDZ+vu9Pr1ZEtlBMyEW8T4aGyM4sw/ZGrfLwtA/V7

uRh/+/mWeoyvNQxx80dQJ3Jy4UIbnHFOBND/4Skg=="}

vp2

{"height":20,"currentBlockHash":"Rdat/CfO/11ULfcjpX+tOTlB76+6YsZwmijdfk7CT8qwF9DMNye25Cz0xAUpDrue

KYM1EyLdS9H+zNR4fWXf/g==","previousBlockHash":"mTJtFgrWkkxRvvoBKNrVG7Mz1C3Kt4VRJHQ4SZLsN/3tcQ

MWM8L7lMsDuXzepVa+r6jSk8JZdkmC1PbMYGoqnw=="}

vp3

{"height":24,"currentBlockHash":"Dcy3znnXDx3C79j+JYtY0K5peVSIXWs/3rCiCo49hmXOboqZPXFrLCKCCAzW6t8

3zwmFFGxyJDL3TUYh8z3Kxg==","previousBlockHash":"G2TYiTDZ+vu9Pr1ZEtlBMyEW8T4aGyM4sw/ZGrfLwtA/V7

uRh/+/mWeoyvNQxx80dQJ3Jy4UIbnHFOBND/4Skg=="}

[vagrant@ubuntu-1404 fabric]$
